# Use of digital retinography to detect vascular changes in pre-diabetic patients: a cross-sectional study

**DOI:** 10.1186/s13098-023-01154-2

**Published:** 2023-11-06

**Authors:** Levimar Rocha Araújo, Juliana Lambert Orefice, Monica Aramuni Gonçalves, Nathalia Sernizon Guimarães, Aleida Nazareth Soares, Tassila Salomon, Alessandra Hubner de Souza

**Affiliations:** 1 Faculdade de Ciências Médicas de Minas Gerais, Belo Horizonte, Minas Gerais Brazil; 2 Ophthalmological Center, Belo Horizonte, Brazil; 3 Metabolica Institute of Applied Medicine, Belo Horizonte, Brazil; 4 Faculdade de Ciências Médicas de Minas Gerais and Faculdade de Saúde Santa Casa BH, Belo Horizonte, Brazil

**Keywords:** Diabetes mellitus, Pre-diabetes, Ophthalmological diagnostic techniques, Digital photograph, Vascular changes, Type 2 diabetes mellitus, Obesity

## Abstract

**Background:**

Diabetic retinopathy (DR) is a common complication of DM and may go unnoticed until irreversible damage occurs. Its screening can contribute to the early detection. Although, there are no studies which investigate the ability of digital retinography to detect vascular changes in pre-diabetic patients.

**Objective:**

Identify the prevalence and severity of RD in patients with pre-diabetes.

**Methods:**

Cross-sectionalstudy carried out in a sample of patients with pre-diabetes and weight excess characterized from January 2020 to April 2023. Sociodemographic and clinical variables were collected, in addition to lifestyle habits. Retinographic evaluation was also performed using a Digital Retinography. For the analysis of all variables, the adopted significance level was 5%. The software used for the analysis was SPSS version 25.0.

**Results:**

Of 108 patients selected 7.1% have alteration in the exam indicating DR. Among the participants with diabetic retinopathy, four had the moderate form (50%), three the moderate form (37%) and only one participant had the severe form (13%).

**Conclusions:**

Our findings highlight the importance of preventive measures and adequate control of these conditions in pre-diabetic patients, in order to prevent or delay the progression of diabetic retinopathy and, consequently, reduce the risk of blindness and other ocular complications.

**Supplementary Information:**

The online version contains supplementary material available at 10.1186/s13098-023-01154-2.

## Introduction

Prediabetes, a condition in which blood glucose values are above reference values, however, below diagnostic values for diabetes mellitus (DM), is positively associated with the risk of all-cause mortality and the incidence of chronic 'diabetes-related' complications, cardiovascular outcomes, stroke, chronic kidney disease, cancer, and dementia [1–3]. In 2021, the U.S. Preventive Services Task Force (USPSTF) recommended prediabetes screening for asymptomatic adults aged 35–70 years with overweight/obesity, lowering the age from 40 years in its 2015 recommendation. The USPSTF suggested considering earlier screening in racial and ethnic groups with high diabetes risk at younger ages or lower Body Mass Index (BMI) [4].

With a global prevalence of 22.3% diabetic retinopathy (DR) remains a common complication of DM and a leading cause of preventable blindness in the adult working population [5]. This is a microvascular complication of diabetes, that can go undetected and unnoticed until irreversible damage even blindness has occurred [6]. The current diagnostic thresholds for diabetes are still based on historic data correlating glycemic parameters with retinopathy; however, an excess prevalence of retinopathy has also been reported in prediabetes. According to the meta-analysis conducted by Kirthi et al. [7], retinopathy prevalence estimates ranged between 0.3 and 14.1% with high variance in estimates due to differing screening methods, retinopathy grading protocols, and study populations.

Understanding that retinal screening contributes to the early detection of diabetic retinopathy and timely treatment, previous studies have investigated digital retinopathy, as a method with high sensitivity and specificity is essential for an effective screening program of digital retinopathy [8, 9]. However, to our knowledge, no study has been investigating the ability of digital retinography to detect vascular changes in pre-diabetic patients.

Given the context presented, the objective of this study was to **i**dentify the prevalence and severity of RD in patients with pre-diabetes.

## Material and methods

A cross-sectional study performed at the “*Instituto Metabólica de Medicina Avançada*”, in Belo Horizonte, Minas Gerais, Brazil, in a convenience sample of patients with pre-diabetes and characterized by HbA1c ≥ 5.6% and ≤ 6.4 in the period from January 2020 to April 2023.

The inclusion criteria were: patients aged 18 years or older, with laboratory test HbA1c lower than 5.7% with 2 or more fasting glucose levels above 126 mg/dL. We did not include patients who already had HbA1c values, greater than 6.4% and/or those with active heart disease including: myocardial infarction, congestive heart failure, angina pectoris, or malignant diseases under treatment or with an interval shorter than 6 months after its completion, pregnant women and those who were diagnosed with autoimmune diseases being treated with corticosteroids and/or immunosuppressant. The sample size consisted of 108 participants.

The sociodemographic variables (gender, age, marital status, and education) were collected using a clinical standardized registration form, as well as the clinical variables: BMI, systemic arterial hypertension (SAH), obstructive sleep apnea, lower limb osteoarthritis, mental disorders, medications in use and pre-existing retinal damage (in case of pre-existing, they were excluded). Life habits (smoking) were also collected.

Education level was defined as Elementary School High School University education. Smoking was categorized into yes and no.

SAH was defined as Blood Pressure (BP) ≥ 140/90 mmHg in two outpatient measurements, according to the American Heart Association (AHA) or the use of antihypertensive medications [10].

### Allocation and procedures

It was performed a retinographic evaluation using a Digital EYER Retinographer (BAYER) with color photos of the fundus of both eyes, without pupillary dilation, taken by a single technician.

The evaluation parameters used were the presence of micro aneurysms, hemorrhages, abnormal arteriovenous crossings, and focal or generalized arteriolar narrowing.

The images of the digital retinography were sent for the elaboration of a report by two ophthalmologists from a specialized reference center in Belo Horizonte. A crossing of the fundus images was carried out between the two professionals with a subsequent comparison of the results. The same-trained technician who photographed the retina and sent it to the specialized center evaluated the patients.

### Ethical aspects

We conducted this study in accordance with resolution 466/2012. This research was submitted to the analysis and appreciation of the Research Ethics Committee (CEP) of *Faculdade Ciências Médicas de Minas Gerais*, according to CONEP regulations, under the number CAAE: 64721722.2.0000.5134. We obtained informed consent from all participants using a procedure approved by the Ethics Committee.

The freedom and integrity of research participants and the preservation of data that could identify them were protected. Privacy and confidentiality were guaranteed. In order to guarantee the confidentiality of the data, the researcher formally committed to the requirement of a prior signature of the commitment form to participate in the project and use of data in files (Additional file 1: Appendix S1).The database was built with nominal identification (only the Capital Letters of the initials of each patient).The researchers involved in the study signed a Term of Commitment for the Use of Data (Additional file 1: Appendix S2) in which they committed themselves to maintain the confidentiality of the data collected in the archives of the bank or collection institution, as well as the privacy of its contents, as recommended by Resolution 466/12, and its complementary ones, of the National Health Council, as well as publishing the paper regardless of the result.

There are no previously established criteria for the interruption of the research, but if by chance the researchers identified a situation that compromised the well-being of the researched or deviated from the objectives, the CEP would be notified of the interruption or suspension of the research before the occurrence of the fact.

The publication of results took place independently of the concluded findings, by publication in a scientific journal or communication at scientific events, with due credit to the authors and institutions. The publication was freely accessible and everyone will be able to see the results only compiled, which guarantees the confidentiality and secrecy of the research participants' data.

### Risks and benefits

The main risks are due to breach of privacy and confidentiality of data. For this reason, the identification of the participants has a medical record number and individual results were compiled for statistical analysis. The right of free will was respected for not answering questions that were not accepted by the researcher or that could cause embarrassment. There were no risks in performing the digital retinography since it was not necessary to dilate the pupil, but there may have been discomfort due to the camera flash in the eyes. There were no direct benefits, but the generated data can add to the understanding of the impacts of early diagnosis of cardiovascular impairment, benefiting those who underwent this approach.

### Statistical analysis

All data were stored in an Excel spreadsheet and within the retinographer's own program. An exploratory analysis of the data was performed to obtain the studied sample characteristics. Categorical variables were described as absolute and relative frequency (%), and continuous variables as mean and standard deviation (SD), minimum and maximum values. Quantitative variables were tested for normality using the Kolmogorov–Smirnov test. The t-test was used for the age variable and the Mann–Whitney test was used for BMI and glycated hemoglobin. For categorical variables, the Chi-square and Chi-square tests with Monte-Carlo simulation were used. In all tests, the significance level adopted was 5%, therefore, comparisons whose p-value was less than or equal to 5% were considered significant. The software used for the analysis was SPSS version 25.0.

## Results

**Fig. 1:**
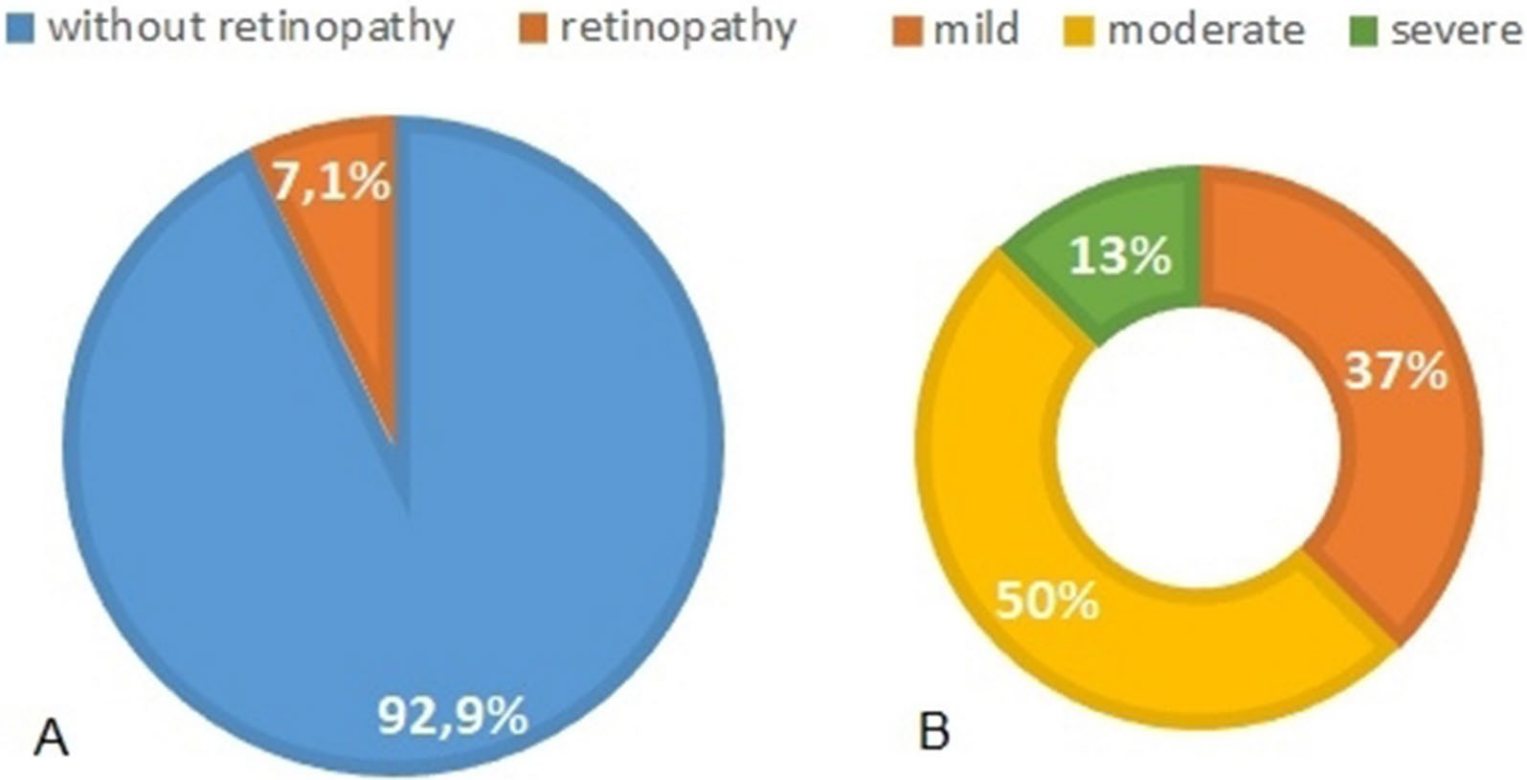
Result of digital retinography and the severity of diabetic retinopathy

Of 108 patients selected, 7.1% have alteration in the exam indicating DR. Among the participants with diabetic retinopathy, four had the moderate form (50%), three the mild form (37%) and only one participant had the severe form (13%). (Fig. 1A and B).

Table 1 presents the sociodemographic, behavioral, and clinical health characteristics of the study participants. Most participants were male (67.9%), had higher education (49.1%) and 52.7% were married. The mean age was 62.6 ± 14.2 years. As for comorbidities, 42% reported high blood pressure, 53.7% had dyslipidemia, 52.7%, and 63.4% were obese. BMI ranged from 17.9 kg/m^2^ to 42.9 kg/m^2^, with a mean of 27.58 ± 5.06 kg/m^2^. Regarding glycated hemoglobin, an average of 5.9 ± 0.3% was observed, with a minimum of 5.7% and a maximum of 7.1%. Most participants were non-smokers (76.8%) (Table 1).

**Table 1:** Distribution of sociodemographic and health variables in a sample of 108 patients with prediabetes

Variables	n (%)
Gender	
Male	76 (67.9)
Female	36 (32.1)
Age (year)	
Mean (SD)	62.6 (14.2)
Min–Max	15–96
Educational level	
Elementary school	5 (4.5)
High school	52 (46.4)
University education	55 (49.1)
Marital status	
Married	59 (52.7)
Separated	6 (5.4)
Single	37 (33)
Widower	10 (8.9)
Comorbidities	
SAH	47 (42)
Dyslipidemia	53 (47.3)
Obesity	71 (63.4)
BMI	
Mean (SD)	27.6 (5.1)
Min–Max	17.9–42.9
Glycated hemoglobin	
Mean (SD)	5.9 (0.3)
Min–Max	5.7–7.1
Smoking	26 (23.2)

Table 2 presents the evaluation of the analyzed variables according to the digital retinography result. When analyzing the associations between demographic variables and the presence of diabetic retinopathy, it was observed that age was significantly higher among participants with diabetic retinopathy (p = 0.015), as well as BMI (p = 0.037).

**Table 2:** Evaluation of the result of digital retinography according to the study variables

Variables	No DR abnormalities n (%)	Retinopathy n (%)	p-value
Gender			
Male	72 (69.2)	4 (50.0)	0.262^q^
Female	32 (30.8)	4 (50.0)	
Age (year)			
Mean (SD)	62 (15.0)	70 (7.0)	**0.015**^**t**^
Min–Max	15–96	61–783	
BMI			
Median	26.5 (23.7–30.7)	30.7 (28.5–31.6)	**0.037**^**mw**^
(P25–P75)			
Min–Max	17.9–42.9	27–34	
Educational level			
Elementary school	5 (4.8)	0 (0.0)	> 0.999^mc^
High school	48 (46.2)	4 (50.0)	
University education	51 (49.0)	4 (50.0)	
Marital status			
Married	55 (52.9)	4 (50.0)	0.371^mc^
Separated	6 (5.8)	0 (0.0)	
Single	35 (33.7)	2 (25.0)	
Widower	8 (7.7)	2 (25.0)	

*mw* Mann–Whitney, *t* t-test, *q* Chi-square, *mc* Chi-square with Monte-Carlo Simulations

When analyzing the comorbidities, smoking, and glycated hemoglobin, it was evidenced that the presence of diabetic retinopathy was significantly associated with the presence of arterial hypertension (SAH) (p = 0.007), dyslipidemia (p = 0.002) and obesity (p = 0.026), all showing a higher percentage for participants with retinopathy. Furthermore, the median value of glycated hemoglobin was higher in patients who had diabetic retinopathy (p = 0.031) (Table 3).

**Table 3:** Evaluation of comorbidities and glycated hemoglobin according to digital retinography results, n = 108

Variables	No DR abnormalities (n = 104) n (%)	Retinopathy (n = 8) n (%)	p-value
SAH			
No	64 (61.5)	1 (12.5)	**0.007**^q^
Yes	40 (38.5)	7 (87.5)	
Dyslipidemia			
No	59 (56.7)	0 (0.0)	**0.002**^q^
Yes	45 (43.3)	8 (100.0)	
Obesity			
No	41 (39.4)	0 (0.0)	**0.026**^q^
Yes	63 (60.6)	8 (100)	
Smoker			
No	82 (78.8)	4 (50.0)	0.063^q^
Yes	22 (21.2)	4 (50.0)	
Glycated hemoglobin			
Median	5.8 (5.7–6.1)	6.1 (6–6.1)	**0.031**^**mw**^
(P25–P75)			
Min–Max	5.7–7.1	5.9–6.1	

*mw* Mann–Whitney, *q* Chi-square, *mc* Chi-square with Monte Carlo simulations

## Discussion

The results suggest that the presence of comorbidities, such as SAH, dyslipidemia and obesity, as well as inadequate glycemic control, represent factors associated with the risk of developing diabetic retinopathy in patients with pre-diabetes. The pathophysiology of DR is complex, but studies demonstrate an association between SAH, a sedentary lifestyle, and poor eating habits with the increase in the global prevalence of DR [11, 12]. Furthermore, it is important to emphasize that the triad, dyslipidemia, arterial hypertension, and high glucose levels is characterized by metabolic syndrome (MS). In general, MS corresponds to the combination of several factors as visceral obesity, lipid disturbances, insulin resistance and hypertension [13, 14]. Several studies indicate a higher risk of development of diabetic retinitis in diabetic patients with metabolic syndrome [15–17]. Its screening, early identification, and initiation of a specific treatment for its various components, such as correct medication intake, and changing lifestyle habits are essential to reduce the complications that compromise the functional and vital prognosis of these patients[15, 18].

Dyslipidemia and obesity variables were impaired in the regression model, as there were no cases for the “no” category in those who had retinopathy, but according to the result of the univariate analysis above, the importance of these variables as an associated factor was perceived. In the final model, only the SAH variable was associated with retinopathy in pre-diabetic patients, but this result cannot be extrapolated to the population.

An attempt was made to build a regression model in order to find the factors that together would be associated with diabetic retinopathy in pre-diabetic patients. It is known that the sample is small for this inference, so it is important to increase the sample size to identify any interesting findings in future work.

In conclusion, our findings highlight the importance of preventive measures and adequate control of these conditions in pre-diabetic patients, in order to prevent or delay the progression of diabetic retinopathy and, consequently, reduce the risk of blindness and other ocular complications.

### Supplementary Information

**Additional file 1**: **Figure 1A**: Result of digital retinography.

**Additional file 2**: **Figure 1B**: Results of diabetic retinopathy severity.

## Data Availability

Not applicable.

## Abbreviations

DM: Diabetes mellitus
DR: Diabetic retinopathy
USPSTF: United States Preventive Services Task Force
BMI: Body mass index
SAH: Systemic arterial hypertension
SD: Standard deviation
MS: Metabolic syndrome
