# Supplementary material for: Use of digital retinography to detect vascular changes in pre-diabetic patients: a cross-sectional study
Source: Diabetol Metab Syndr. 2023 Nov 6;15:225. doi: 10.1186/s13098-023-01154-2 (PMC10626765; doi:10.1186/s13098-023-01154-2)
Supplement: Supplementary file 1 — Additional file 1: Figure 1A: Result of digital retinography. [file 13098_2023_1154_MOESM1_ESM.pdf]

TERMO DE CONSENTIMENTO LIVRE ESCLARECIDO

Estudo clínico para detecção de Diabetes mellitus tipo 2 por retinografia digital

Nome dos Responsáveis: Tassila Salomon, Levimar Rocha Araújo e Alessandra Hubner de Souza

Nós estamos convidando você para participar como voluntário da pesquisa "Estudo clínico para detecção de Diabetes mellitus tipo 2 por retinografia digital."

Este documento tem o objetivo de lhe dar informações sobre a pesquisa e de assegurar seus direitos como participante da pesquisa. Por favor, antes de decidir participar da pesquisa, leia este Termo com calma e atenção, use o tempo que precisar. Se você tiver dúvidas pode perguntar a qualquer momento. Se preferir, pode levar este Termo para casa e consultar seus familiares, amigos ou outras pessoas antes de decidir participar da pesquisa.

Primeiro nós queremos que você saiba que sua participação nesta pesquisa é opcional. Você pode decidir não participar da pesquisa ou, caso decida participar, você pode sair dela a qualquer momento, sem penalização ou prejuízo para você. Você pode fazer perguntas a qualquer momento.

Depois que você entender a pesquisa e concordar em participar, nós iremos te pedir para assinar ou colocar a sua impressão digital neste documento. Este documento será elaborado em duas vias, uma será dada para você guardar e outra via será arquivada pelo pesquisador. As duas vias serão assinadas por você e pelo responsável da pesquisa.

JUSTIFICATIVA E OBJETIVOS

Por que estamos fazendo esta pesquisa?

O motivo que nos leva realizar esta pesquisa para tentar detectar e compreender as alterações vasculares à retinografia digital (um tipo de exame de imagem), anteriores ao diagnóstico laboratorial de Diabetes Mellitus tipo 2 (DM2). O objetivo da pesquisa é identificar os fatores de risco para o desenvolvimento da DM2 com a história clínica, ao exame físico com as medidas antropométricas, exames laboratoriais e de imagem.

Por que estamos convidando você para fazer parte desta pesquisa?

Estamos te convidando para fazer parte dessa pesquisa, pois você faz tratamento no Hospital Universitário da Ciências Médicas de Minas Gerais ou na Clinica Metabólica, em Belo Horizonte, Minas Gerais e exames laboratoriais compatíveis com nossos critérios de inclusão na

pesquisa (exame laboratorial prévio de HbA1c entre os níveis 5,7% e 6,4% e obesidade estágio 2 ou mais).

PROCEDIMENTOS

Como será sua participação nesta pesquisa?

Se você concordar em participar desta pesquisa, um profissional treinado irá realizar um exame em você, uma avaliação retinográfica por meio de Retinógrafo Digital) com realização de fotos coloridas do fundo de olho de ambos os olhos, sem a dilatação pupilar. O exame dura em média de 10 minutos. Também faremos algumas perguntas sobre seu histórico médico e seus hábitos de vida.. O exame será realizado uma única vez em cada paciente, que serão selecionados pelo Dr. Levimar durante sua consulta na Metabólica Instituto, e por isso não terão necessidade de deslocamento ou despesas extras. O questionário será preenchido durante a execução do exame pelo próprio técnico que fara. As informações não serão gravadas, somente armazenadas em arquivo profissional da clínica durante o período de 10 anos após término do projeto.

DESCONFORTOS, RISCOS E BENEFÍCIOS

Existe algum desconforto ou risco para você por participar desta pesquisa?

Os principais riscos se devem a quebra de sigilo e confidencialidade dos dados. Para isso a identificação dos participantes terá número do prontuário e os resultados individuais serão compilados para análise estatística. Você pode optar por não responder qualquer pergunta, caso não queria ou que possa de deixar constrangido.

Existe algum benefício para você por participar desta pesquisa?

Não existem benefícios diretos, mas os dados gerados por esta pesquisa poderão nos ajudar a compreender os impactos do diagnóstico precoce de comprometimento cardiovascular beneficiando à comunidade que necessita de atendimento.

ACOMPANHAMENTO E ASSISTÊNCIA

Você pode nos comunicar a qualquer momento, tendo meu telefone à disposição ou comparecendo ao ambulatório onde é feita a pesquisa. Meu telefone 31 984814773 (Tassila – pesquisadora principal do estudo). O local da pesquisa é no Hospital Universitário da Ciências Médicas de Minas Gerais e a Clinica Metabólica, em Belo Horizonte, Minas Gerais.

RESSARCIMENTO E INDENIZAÇÃO

Você receberá pagamento por participar desta pesquisa?

Você não receberá nenhum pagamento, em dinheiro ou em outra forma, por participar desta pesquisa.

<u>Você terá algum custo participando desta pesquisa?</u>

A sua participação nesta pesquisa não acarretará custos a você. Se você tiver que pagar para participar da pesquisa, com despesas como alimentação ou transporte, ou tiver qualquer outra despesa devido a esta pesquisa, você e seu acompanhante (caso este seja necessário), serão ressarcidos.

<u>O que acontece se eu tiver algum dano por causa desta pesquisa?</u>

Se você sofrer algum dano **decorrente** desta pesquisa, você tem direito à assistência integral e gratuita sem qualquer restrição ou condicionante, assim como o direito a indenização.

GARANTIA DE SIGILO E PRIVACIDADE

<u>Seus dados e suas informações serão mantidos em segredo?</u>

Você tem a garantia de que sua identidade será mantida em sigilo e que nenhuma informação será dada a outras pessoas que não façam parte da equipe de pesquisadores. Seu nome ou o material que indique a sua participação não será liberado sem a sua permissão. Na divulgação dos resultados dessa pesquisa, seu nome não será citado. Os dados coletados terão sigilo garantido pelos autores responsáveis. Todas as informações deste estudo são confidenciais. Os dados pessoais dos participantes só serão fornecidos se requeridos por lei. Seu nome não será publicado na hora de divulgar os resultados. A equipe de pesquisadores poderá ter acesso aos seus registros. Esse acesso será utilizado para realizar, acompanhar a pesquisa e analisar os dados obtidos.

As normas brasileiras que protegem os participantes voluntários de pesquisa serão respeitadas. Os dados podem ser publicados.

CONTATO

<u>Quem você poderá contatar se tiver perguntas?</u>

Você pode fazer perguntas ou solicitar novas informações em qualquer momento da pesquisa.

PARA ESCLARECER DÚVIDAS SOBRE A PESQUISA você deve entrar em contato com o pesquisador e sua equipe.

Pesquisador Principal: Tassila Salomon. Rua Vênus, 505/403. Ana Lucia, Sabará. Telefone: 31 984814773. Email: tassila.sangy@cienciasmedicasmg.edu.br

Outro membro da equipe: Levimar Rocha Araújo**,** no telefone 31 9972-6262 ou Alessandra Hubner de Souza, no telefone 51 98237-9903.

PARA ESCLARECER DÚVIDAS SOBRE OS SEUS DIREITOS COMO PARTICIPANTE DA PESQUISA você deve entrar em contato com o **Comitê de Ética em Pesquisa das Ciências Médicas (CEPCM-MG).** O Comitê de Ética em Pesquisa (CEP) é um colegiado composto por pessoas voluntárias, com o objetivo de defender os interesses dos participantes da pesquisa em

sua integridade e dignidade e para contribuir no desenvolvimento da pesquisa dentro de padrões éticos. O CEPCM-MG é diretamente vinculado à Faculdade de Ciências Médicas de Minas Gerais e outros institutos mantidos pela Fundação Educacional Lucas Machado. Você também pode fazer denúncias ou reclamações sobre sua participação e sobre questões éticas do estudo.

Comitê de Ética em Pesquisa das Ciências Médicas (CEPCM-MG):

Endereço: Alameda Ezequiel Dias, nº 275, Bairro Centro. CEP: 30130-110 - Belo Horizonte /MG. *Telefone:* (31) 3248-7155. *Horário de funcionamento:* 09h às 18h

E-mail: cep@feluma.org.br

CONSENTIMENTO LIVRE E ESCLARECIDO

Após ter lido, discutido e entendido este Termo de Consentimento; após ter recebido esclarecimentos sobre o motivo da pesquisa, seus objetivos, procedimentos, benefício, potenciais riscos e incômodos que esta possa acarretar a você; após todas as suas dúvidas serem esclarecidas, se aceitar participar da pesquisa, por gentileza, preencha os campos abaixo.

Será fornecido a você uma via original deste documento assinada pelo pesquisador e por você, tendo todas as folhas por nós rubricadas.

Nome Legível do participante: ____

Nome Legível do responsável legal do participante, quando necessário: ____

Telefone: ____

E-mail (opcional): ____

Data: ____/_____/______.

Assinatura do participante ou Responsável legal: ______________________________

RESPONSABILIDADE DO PESQUISADOR

Asseguro ter cumprido as exigências da resolução 466/2012 do CNS/MS e complementares na elaboração do protocolo e na obtenção deste Termo de Consentimento Livre e Esclarecido. Asseguro ter sanado todas as dúvidas do participante da pesquisa. Declaro ter fornecido uma via original deste documento assinada pelo participante e por mim, tendo todas as folhas por nós rubricadas. Informo que o estudo foi aprovado pelo CEPCM-MG e pela CONEP, quando aplicável. Comprometo-me a utilizar o material e os dados obtidos nesta pesquisa exclusivamente para as finalidades previstas neste documento ou conforme o consentimento dado pelo participante.

Data: ____/____/_____.

____

Assinatura do pesquisador responsável

Tassila Salomon
