# Supplementary material for: Use of digital retinography to detect vascular changes in pre-diabetic patients: a cross-sectional study
Source: Diabetol Metab Syndr. 2023 Nov 6;15:225. doi: 10.1186/s13098-023-01154-2 (PMC10626765; doi:10.1186/s13098-023-01154-2)
Supplement: Supplementary file 2 — Additional file 2: Figure 1B: Results of diabetic retinopathy severity. [file 13098_2023_1154_MOESM2_ESM.docx]

**TERMO DE COMPROMISSO PARA USO DE DADOS EM ARQUIVO**

**Título do projeto:** Estudo clínico para a detecção de diabetes mellitus tipo 2, por retinografia digital

**Pesquisadores: Levimar Rocha Araújo, Tassila Salomon, Alessandra Hubner de Souza**

O(s) pesquisador(es) do projeto acima identificado(s) assume(m) o compromisso de:

1. Preservar a privacidade dos participantes cujos dados serão coletados;
2. Que as informações utilizadas única e exclusivamente para a execução do projeto em questão;
3. Que as informações somente serão divulgadas de forma anônima, não sendo usadas iniciais ou quaisquer outras indicações que possam identificar o participante da pesquisa.
4. Que os pesquisadores só poderão fazer uso do material de coleta de dados (prontuários) da base nas dependências da Instituição Pesquisada, sendo absolutamente vedada a saída de arquivos ou prontuários, sob qualquer forma, das dependências da Instituição.
5. Que serão respeitadas todas as normas da Resolução 466/12 e suas complementares na execução deste projeto.

Belo Horizonte, 8 Julho de 2022

___________________________________________

Tassila Salomon

**Pesquisador Responsável**

___________________________________________

**Levimar Rocha Araújo**

___________________________________________

**Alessandra Huber de Souza**
